# Occurrence and risk characterization of aflatoxin M_1_ in milk samples from southeastern Iran using the margin of exposure approach

**DOI:** 10.1002/fsn3.3634

**Published:** 2023-08-25

**Authors:** Ali Ghaffarian‐Bahraman, Salman Mohammadi, Ali Dini

**Affiliations:** ^1^ Occupational Environment Research Center Rafsanjan University of Medical Sciences Rafsanjan Iran; ^2^ Department of Nutrition, School of Health and Nutrition Lorestan University of Medical Sciences Khorramabad Iran; ^3^ Pistachio Safety Research Center Rafsanjan University of Medical Sciences Rafsanjan Iran

**Keywords:** aflatoxin‐M1, HPLC, margin of exposure, Milk

## Abstract

This study aimed to investigate Aflatoxin‐M_1_ (AFM_1_) contamination in pasteurized and raw milk samples consumed in Kerman and Rafsanjan in southeastern Iran. In this cross‐sectional study, a total of 100 samples of raw (*n* = 67) and pasteurized (*n* = 33) milk were randomly collected from retail stores, supermarkets, and milk transport tankers in the winter of 2020 and the summer of 2021. The level of AFM_1_ contamination in the collected samples was evaluated by high‐performance liquid chromatography with fluorescence detection (HPLC‐FD). AFM_1_ was detected in 95% of samples and its median concentration was 17.38 ng/L. The median concentration of AFM_1_ in the pasteurized milk samples (24.89 ng/L) was significantly higher than in the raw milk samples (13.54 ng/L). The AFM_1_ contamination level in 20% (raw = 13% and pasteurized = 7%) of the samples was higher than the maximum permitted level (MPL) recommended by the European Union (i.e., 50 ng/L), whilst 4% (raw = 3% and pasteurized = 1%) of the samples was higher than the Iranian maximum standard limit (i.e., 100 ng/L). The hazard index (HI) was higher than 1 in 16%, 18%, and 35% of total milk samples for men, women, and children, respectively. The AFM_1_ contamination level in the milk samples collected in southeastern Iran was worrying. The margin of exposure (MoE) values were lower than 10,000 for children. Because aflatoxins are among the most potent carcinogens known, prevention of milk contamination in all stages from the farm to the table can considerably reduce the community's exposure to AFM_1_ and its consequent health risks.

## Graphical abstract

This study aimed to investigate Aflatoxin‐M1 contamination in pasteurized and raw milk samples consumed in southeastern Iran. AFM1 was detected in 95% of the analyzed samples. The hazard index (HI) of AFM1 exposure was in the warning range in 16%, 18%, and 35% of milk samples for men, women, and children, respectively. The results demonstrated that margin of exposure (MoE) values were lower than 10,000 for children. The AFM1 contamination level in the milk samples collected in southeastern Iran was worrying.

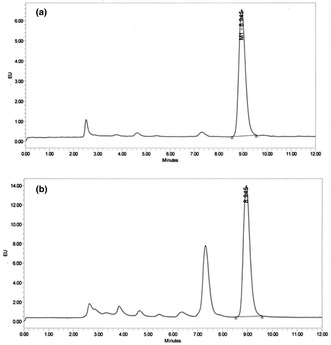

## 1 INTRODUCTION

Globally, biological and chemical contaminants in food and feed are serious threats to the health of humans and animals (Jafari et al., 2012; Kebede et al., 2020). Aflatoxins are the most well‐known group of mycotoxins that are mainly produced by *Aspergillus flavus* and *Aspergillus parasiticus* (Ismail et al., 2018). Among aflatoxins, types B1, B2, M1, G1, and G2 are more important with respect to food hygiene and safety (Corassin et al., 2022; Zain, 2011). If lactating animals are fed aflatoxin‐B1 (AFB1) contaminated feeds, the toxin is metabolized in the animals' liver and converted to AFM_1_ to appear in the animal's milk, urine, and feces as one of the excretory metabolites (Nguyen et al., 2020). Studies have shown that there is a direct relationship between the concentration of AFM_1_ in the milk and AFB1 in the feeds of animals so that between 1% and up to 6% of AFB1 consumed by livestock is excreted in the form of AFM_1_ through milk (Britzi et al., 2013; Masoero et al., 2007). Factors that have been shown to affect the carry‐over rate were species difference, amount of milk yield, general health of the animal, hepatic biotransformation capacity, and rate of ingestion (Britzi et al., 2013).

AFB1 has shown health‐threatening effects including toxic, teratogenic, mutagenic, and carcinogenic properties, since it is known as the most hazardous type of aflatoxin (EFSA Panel on Contaminants in the Food Chain et al., 2020; Iqbal et al., 2015). Although the mutagenicity of AFM_1_ is far lower than that of AFB1, its exposure can also cause cytotoxicity and DNA damage in human's body (Zhang et al., 2015). It is shown that aflatoxins' exposure has occurred in 2.6–28.2% of all global hepatocellular carcinoma cases (Liu & Wu, 2010). In addition, a significant correlation has been observed between the level of aflatoxins' excretory metabolites in urine (including AFM_1_) and hepatocarcinoma incidence in hepatitis‐B virus‐positive individuals (Sun et al., 1999). The International Agency for Research on Cancer (IARC) classifies AFB1 as carcinogenic to humans (Group 1) and AFM_1_ as possibly carcinogenic to humans (Group 2B) (IARC, 2012; Ostry et al., 2017).

AFM_1_ can be detected in a variety of dairy products due to its stability against processes such as pasteurization and sterilization (Mohammadi et al., 2021; Mohammadi, Behmaram, et al., 2022).

There are three methods usually used for the quantification of AFM_1_ in dairy products including the enzyme‐linked immunosorbent assay (ELISA), thin layer chromatography (TLC), and HPLC (Vaz et al., 2020). It is difficult to produce an AFM_1_‐free dairy product. Therefore, a maximum permissible limit is introduced for the concentration of this toxin in dairy products in most countries to protect consumers from the dangers of AFM_1_ exposure, especially infants and children. The maximum permissible limit of AFM_1_ in milk is 50 and 500 ng/L in the European Union and the US, respectively (Administration, 2000; Commission, 2006b). However, the maximum standard allowance of AFM_1_ concentration in all types of milk samples in Iran is defined as 100 ng/L (Hashemi, 2016).

Given the widespread use of milk and dairy products as a nutritious and health‐promoting food, measurement of residual toxins of these products is one of the most important challenges in developed and developing societies. In this study, we aimed to examine the contamination level of AFM_1_ in raw and pasteurized milk samples distributed in southeastern Iran and determine the risk of exposure to AFM_1_ in this area.

## 2 MATERIALS AND METHODS

### 2.1 Sampling design

The process of random milk sampling (raw and pasteurized) was conducted in the winter of 2020 and the summer of 2021 in the cities of Rafsanjan and Kerman located in southeastern Iran. Raw milk samples were collected from the supply level (*n* = 43) and transferred tanks carrying milk (*n* = 24) to the processing plants, whilst pasteurized milk samples (*n* = 33) were collected from supermarkets and retail suppliers. The collected samples were transferred to the laboratory and stored at −20°C until the AFM_1_ quantification processes were performed within a maximum of 7 days from the sampling date.

### 2.2 Materials and equipment

The method of reverse phase HPLC (Waters, Binary pump, model 1525, USA) equipped with degassing device (DG2, USA) and Fluorescence detector (Blue, Model 2475, USA) was used to measure the concentration of AFM_1_ in milk samples. A C18 HPLC column (Nova‐Pak, Waters USA) was used as the stationary phase. The mobile phase used included acetonitrile (Merck, Germany): deionized water: methanol (Merck, Germany) in a proportion of 60:20:20 (v/v/v), and the flow rate of the mobile phase was 0.8 mL/min. The column temperature was 35°C and the wavelengths of emission and excitation were set at 435 and 365 nm, respectively.

In order to evaluate the performance quality of the method, validation parameters including sensitivity, accuracy, precision, and linearity of the case were calculated. Sensitivity was expressed as the values of limit of detection (LOD) and limit of quantity (LOQ) defined as the signal‐to‐noise ratio of 3:1 and 10:1, respectively. The accuracy of the method was assessed by analyzing the artificially contaminated milk samples with AFM_1_ at the levels of 0.01, 0.05, and 0.1 ng/mL. The accuracy of the method was also analyzed using FAPAS, Food Analysis Performance Assessment Scheme, samples. FAPAS supply real matrix test samples for all routine analytical disciplines in the fields of food chemistry, food microbiology, genetically modified materials, drinking water chemistry and microbiology, environmental chemistry, and microbiology. All of the analyses were performed in six replications for each level of contamination.

The repeatability of the method was assessed by calculating the recovery rate of each concentration spike in repeated injections (six samples per day) and the reproducibility of the method was evaluated by analyzing the recovery rate of the concentration spikes attributed to the same samples in four different days (*n* = 24). An 8‐point calibration curve was plotted using the AFM_1_ standard solution (0.5 mg/L, Sigma Aldrich, USA) in a concentration range of 0.003 to 4 ng/mL with a three‐replication per concentration. The linearity of the calibration curve was evaluated by linear regression analysis and expressed as the square correlation coefficient (*R* ^2^).

### 2.3 AFM_1_ extraction

The frozen samples were thawed at 37°C and their fat was separated by centrifugation at 4000*g* for 15 min. Then, 50 mL of the defatted samples was passed through an immunoaffinity column with AFM_1_‐specific antibodies, and the column was washed with 20 mL of deionized water. The AFM_1_ accumulated in the column was then extracted using 1.25 mL of acetonitrile solvent and collected in a vial. After diluting the contents of the vial with 1.25 mL of deionized water, 100 μL of the final solution was injected into the HPLC device.

### 2.4 Risk assessment

The risk of exposure to AFM_1_ through milk consumption was calculated using the following equation:

$$\mathrm{DI}\;\left(\mathrm{ng}/\mathrm{kg}-\mathrm{bw}/\mathrm{day}\right)=\frac{\mathrm{DMI}\times C}{\mathrm{BW}}$$

In this formula, DMI is the daily milk consumption (L/day), C refers to the AFM_1_ concentration in milk samples (ng/L), and BW stands for the average body weight of consumers (kg). According to previous studies, the average body weight of Iranian men, women, and 6‐ to 12‐year‐old children was 79.6, 70, and 30 kg, respectively (Aalipour et al., 2015; Ghaffarian Bahraman et al., 2020). The estimated hazard index (HI) of AFM_1_ exposure through milk intake was also calculated based on the daily intake (DI) and tolerable daily intake (TDI) for AFM_1_ as follows:

$$\mathrm{HI}=\frac{\mathrm{DI}\;\left(\mathrm{ng}/\mathrm{kg}-\mathrm{bw}/\mathrm{day}\right)}{\mathrm{TDI}\;\left(\mathrm{ng}/\mathrm{kg}-\mathrm{bw}/\mathrm{day}\right)}.$$

The TDI for AFM_1_ was considered 0.2 based on the previous studies. HI levels above 1 indicate a significant health risk of exposure to AFM_1_ in the milk‐consuming population (Kuiper‐Goodman, 1990).

### 2.5 Risk characterization

The risk of AFM_1_ oral exposure was described by the margin of exposure (MoE) approach proposed by the European Food Safety Authority (EFSA). In fact, MoE is an index to evaluate the risk of oral exposure to compounds with carcinogenic and genotoxic properties (Dini et al., 2022). The value of MoE was calculated using the following equation (Mohammadi, Keshavarzi, et al., 2022).

$$\mathrm{MOE}=\frac{{\text{BMDL}}_{10}}{\mathrm{DI}}$$

Here, BMDL_10_ is a benchmark dose of the toxic substance that increases the incidence of liver cancer (HCC = hepatocellular carcinoma) by 10%. A value of MoE <10,000 reflects a worrying risk level of HCC in the community induced by AFM_1_ exposure. Based on studies in animals, a BMDL_10_ of 0.4 μg/kg bw per day for the incidence of HCC in male rats following AFB1 exposure is to be used in MOE approach. Since BMDL_10_ level is not determined for AFM_1_, the EFSA approved to use a potency factor of 0.1 in combination with the BMDL_10_ of 0.4 μg/kg bw per day for the induction of HCC by AFB1 for the AFM1 risk assessment. (Chain et al., 2020). Hence, in the present study, a BMDL_10_ of 4 μg/kg bw per day was used for the AFM1 risk assessment.

### 2.6 Hepatocellular carcinoma in human societies

People with hepatitis B virus (HBV) infection appear to have the highest risk of developing HCC due to exposure to AFs. In this study, considering the synergistic effect of exposure to AFM_1_ and HBV on HCC development, the following formula was used to estimate the risk of HCC $\left(\text{cancer cases}/\text{year}/100,000\;\text{Persons}\right)$due to AFM_1_ exposure in the community:

Population risk=RLC×EDI

Where RLC is the risk of liver cancer incidence in the Iran population. According to a recent EFSA report, AFM_1_ carcinogenic potency (CP) in healthy was 0.0017 (mean) and 0.0049 (95% upper bound) cancer cases/year/100000 persons per ng/kg bw/day. Furthermore, CP of AFM_1_ in HBV patients was 0.0269 (mean) and 0.0562 (95% upper bound) cancer cases/year/100000 persons per ng/kg bw/day (Chain et al., 2020). Moreover, it is shown that HBV prevalence in the Iranian population is 2.1% (Razavi‐Shearer et al., 2018). Therefore, the RLC rate was estimated according to the prevalence of HBV in the Iranian population as follows:

$$\mathrm{RLC}=\left(A\times B\right)+\left(\left(1-A\right)\times D\right)$$

Where A is the prevalence of HBV patients in the Iran population (0.021), B is CP of AFM1 in HBV patients, and D is CP of AFM1 in healthy population.

### 2.7 Statistical analysis

Data were cleaned up before analysis and Shapiro–Wilk statistical test was used to assess the normality of quantitative variables. The between‐group comparisons were conducted using the nonparametric statistical test of the two‐sample Wilcoxon rank‐sum (Mann–Whitney *U*) test. Data were analyzed with Stata statistical software version 14.0 (Stata Corp LLC). In the present study, a *p*‐value <.05 was considered significant.

## 3 RESULTS

### 3.1 Sampling and recovery of AFM_1_

**FIGURE 1:**
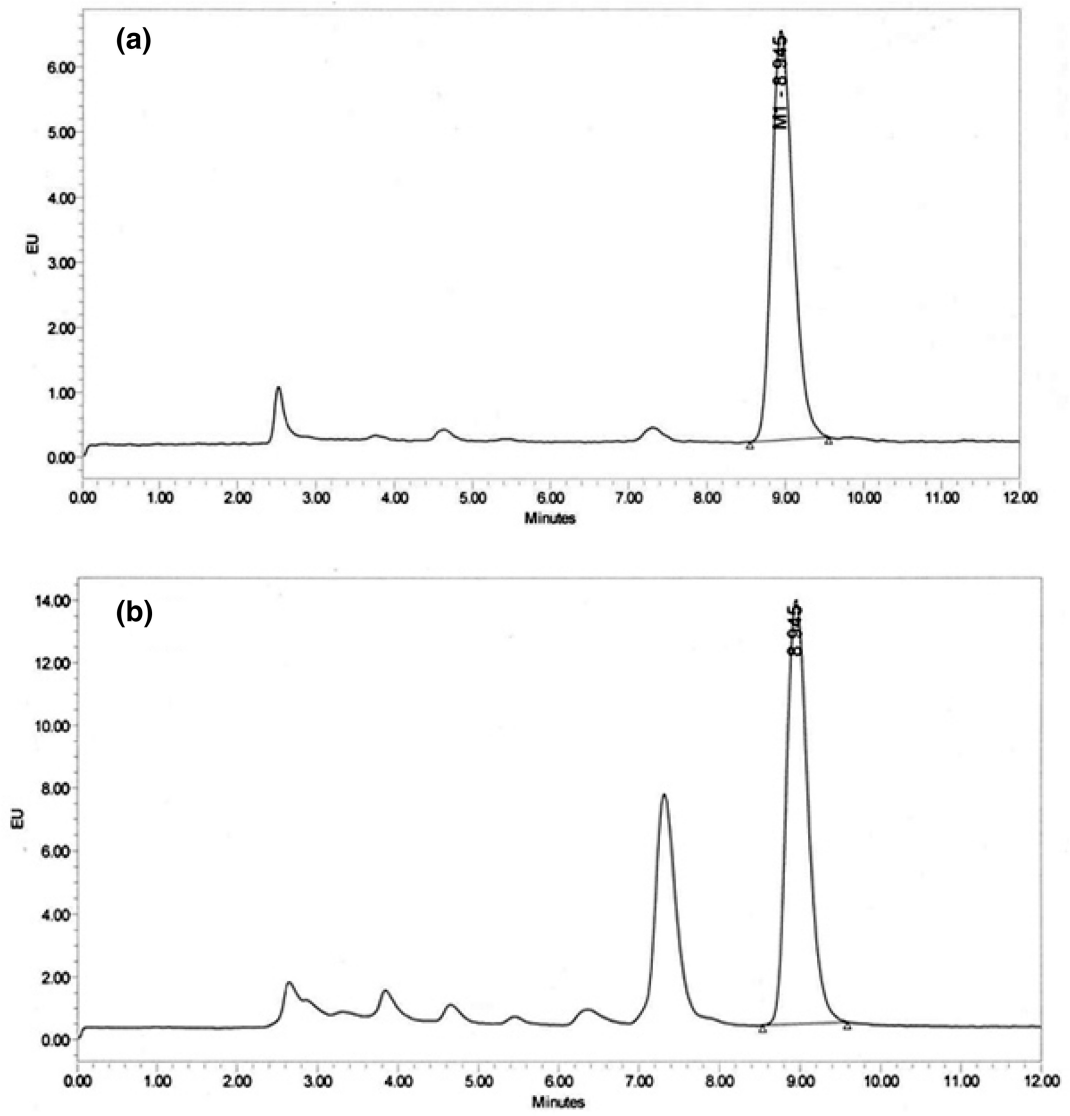
HPLC chromatograms of an artificially contaminated cow milk sample (a); A naturally contaminated cow milk sample (b).

A total of 100 samples of cow milk (33 pasteurized and 67 raw samples) were randomly collected from the cities of Rafsanjan and Kerman located in southeastern Iran. Moreover, the collected samples were categorized into three groups: pasteurized milk (*n* = 33), raw milk of the supply level (*n* = 43), and raw milk collected from transfer tanks (*n* = 24). Detection and quantification of AFM_1_ in milk samples were performed with acceptable accuracy. As demonstrated in the chromatograms of Figure 1, no interference peak was detected near the AFM_1_ retention time (8.94 min). Also, the AFM_1_ curve of calibration was linear in the concentration of 0.02 to 4 ng/mL with an *R* ^2^ value greater than 0.999. The values of LOD and LOQ were 0.7 and 2 ng/L, respectively. The recovery value of AFM_1_ was set in the range of 79.8%–102% (Table 1) complying with EC‐No. 401/2006 (Commission, 2006a).

**TABLE 1:** Recovery percentage of aflatoxin‐M1 in milk samples.

Spiked level (ng/mL)	Repeatability (*n* = 6)								Reproducibility	
	Day 1		Day 2		Day 3		Day 4		Within laboratory (*n* = 24)	
	Mean recovery (%)	RSD (%)	Mean recovery (%)	RSD (%)	Mean recovery (%)	RSD (%)	Mean recovery (%)	RSD (%)	Mean recovery (%)	RSD (%)
0.01	101	6.01	102	4.76	92.5	6.03	93.6	4.56	97.27	6.71
0.05	91	5.86	84.3	7.06	89.2	4.40	93.2	4.26	89.42	6.30
0.1	79.8	6.11	92.3	3.73	83.2	4.26	85.7	4.53	85.3	7.01

Abbreviation: RSD, Relative standard deviations for repeatability.

### 3.2 Concentration and occurrence of AFM_1_ in milk samples

The results of the present study showed that the overall median (interquartile range, or IQR) of AFM_1_ concentration in the milk samples was 17.38 (7.24, 43.08) ng/L. This value was significantly higher in pasteurized milk samples (24.89, 31.34 ng/L) than in raw milk samples (13.54, 35.38 ng/L). However, no statistically significant difference was observed between the raw milk's subgroups, or in comparisons based on cities of origin and season of sampling in terms of AFM_1_ concentration (Table 2). Moreover, we observed that 95% of the samples were AFM_1_ positive and the concentration of AFM_1_ in 20% and 4% of the samples was higher than the maximum permissible limit (MPL) recommended by the European Union (the EU‐MPL, i.e., 50 ng/L) and the Iranian Standards Organization (the ISO‐MPL, i.e., 100 ng/L), respectively. As shown in Table 3, the AFM_1_ mean concentration in two out of 11 studied pasteurized milk brands was higher than the EU‐MPL (brands 10 and 11).

**TABLE 2:** Aflatoxin‐M1 contamination in milk samples.

Outcomes	Total samples	Positive samples *N* (%)	Samples exceeding EU‐MPL^a^, *N* (%)	Samples exceeding ISO‐MPL^b^, *N* (%)	Median^c^ (Q1, Q3) (ng/L)	Min–Max (ng/L)
Milk type						
Raw						
Market	43	39 (90.7)	5 (11.63)	0	14.67 (5.04, 36.52)^d^	<LOQ – 93.7
Bulk tank	24	23 (95.8)	8 (33.33)	3 (12.5)	9.36 (6.65, 71.37)	2.70–189.9
Overall	67	62 (92.5)	13 (19.4)	3 (4.48)	13.54 (5.26, 40.64)^d^	2.02–189.8
Pasteurized	33	33 (100)	7 (21.2)	1 (3.3)	24.89 (14.34, 45.68)	5.40–125.37
City						
Kerman	47	46 (97.8)	13 (27.66)	3 (6.38)	23.21 (7.68, 54.99)	2.75–189.81
Rafsanjan	53	49 (92.45)	7 (13.2)	1 (1.89)	16.02 (6.72, 36.52)	2.02–125.37
Season						
Winter	55	54 (98)	11 (20)	3 (5.45)	14.67 (6.72, 45.52)	2.02–189.81
Summer	45	41 (91.1)	9 (20)	1 (2.22)	18.98 (11.54, 40.64)	2.75–125.37
Overall	100	95 (95)	20 (20)	4 (4)	17.38 (7.24, 43.08)	2.02–189.8

Abbreviations: AFM_1,_ Aflatoxin M_1_; *N*, Number of samples; Q1, First quartile; Q3, Third quartile.

^a^ The maximum permissible limit (MPL) recommended by the European Union (EU‐MPL) for AFM_1_ in milk is 50 ng/L.

^b^ The MPL recommended by the Iranian Standards Organization (ISO‐MPL) for AFM_1_ in milk is 100 ng/L.

^c^ Two‐sample Wilcoxon rank‐sum (Mann–Whitney) test was used for between‐group comparisons.

^d^ Significant differences (*p* < .05) between the Pasteurized and other groups at *α* level of .05.

**TABLE 3:** AFM_1_ concentration in different brands of pasteurized milk samples.

Commercial brand	Sample size (*n*)	AFM_1_ concentration (μg/L)^a^
Brand −1	3	13.66 ± 3.18
Brand −2	3	16.63 ± 14.49
Brand −3	3	21.56 ± 13.40
Brand −4	3	22.35 ± 15.82
Brand −5	3	25.95 ± 20.52
Brand −6	3	29.16 ± 22.58
Brand −7	3	29.55 ± 9.43
Brand −8	3	35.88 ± 13.87
Brand −9	3	48.93 ± 31.87
Brand −10	3	58.61 ± 40.52
Brand −11	3	99.61 ± 22.32

Abbreviation: AFM_1_, Aflatoxin M_1_.

^a^ Data are presented in mean (SD).

### 3.3 Risk assessment

The risk of exposure to AFM1 through milk consumption was characterized using HI, MoE, and the HCC risk approach (Table 4). The HI was calculated using median, Q1 and Q3 levels of AFM_1_ concentration in milk samples was <1 for adults and children. Nevertheless, our results showed that a percentage of the milk samples studied were in the alert range in terms of AFM_1_ contamination level for men (16%), women (18%), and children (35%), based on HI value interpretation (Table 4). The results demonstrated that MoE values were lower than 10,000 for children. Furthermore, the overall MoE for males and females were 14,833 and 13,044, respectively.

**TABLE 4:** Risk assessment of AFM_1_ exposure through milk consumption in southeastern Iran.

	Male median (Q1, Q3)	Female median (Q1, Q3)	Child median (Q1, Q3)
EDI (ng/kg.bw/day)	0.054 (0.023–0.134)	0.061 (0.026–0.152)	0.119 (0.05–0.295)
HI	0.27 (0.112–0.668)	0.307 (0.128–0.760)	0.595 (0.248–1.475)
MoE	14,833 (35609–5984)	13,044 (31315–5262)	6719 (16131–2710)
HCC risk (mean)^a^	0.0006 (0.0003–0.0015)	0.0007 (0.0003–0.0017)	0.0013 (0.0006–0.0033)
HCC risk (upper bound)^b^	0.0016 (0.0007–0.004)	0.0018 (0.0008–0.0045)	0.0036 (0.0015–0.0088)
Samples with HI ≥1 (%)	16	18	35
Samples with MoE ≤ 10,000 (%)	4	4	18

Abbreviations: EDI, Estimated Dietary Intake; HCC, Hepatocellular carcinoma; HI, Hazard Index; MoE, Margin of Exposure; Q1, First quartile; Q3, Third quartile.

^a^ Cancer risk estimates calculated based on the mean of AFM1 carcinogenic potency.

^b^ Cancer risk estimates calculated based on the upper bound of AFM1 carcinogenic potency.

Also, the MoE value calculated for individual samples indicated that 4%, 4%, and 18% of the milk samples were in the alert range for men, women, and children, respectively (Table 4). Based on the mean potency estimates, the HCC risk of exposure to medium levels of AFM_1_ through milk consumption is the cause of 0.0006, 0.0007, and 0.0013 cases of HCC per 100,000 persons per year in men, women, and children, respectively. Based on the upper bound (UB) potency estimates, the cancer risk of exposure to medium levels of AFM_1_ is the cause of 0.0016, 0.0018, and 0.0036 cases of HCC per 100,000 persons per year in men, women, and children, respectively (Table 4).

## 4 DISCUSSION

Today, aflatoxin contamination of food items has become an issue of concern worldwide. We observed in the current study that 95% (*n* = 95) of the overall milk samples collected in the southeastern area of Iran were contaminated with AFM_1_, whilst this value was 92.5% (*n* = 62) and 100% (*n* = 100) in raw and pasteurized milk samples, respectively. In addition, the AFM_1_ contamination level was found to exceed the EU‐MPL (50 ng/L) in 19.4% (*n* = 13) of raw samples and 21.2% (*n* = 7) of pasteurized samples.

The occurrence of AFM_1_ contamination in milk samples has been previously reported in Iran and other countries. A recent meta‐analysis showed that the average prevalence of AFM_1_‐positive milk samples collected in the different areas of Iran was 82%, whilst it was various for raw (72%), sterilized (90%), and pasteurized milk samples (96%). According to the above meta‐analysis, the prevalence of the EU‐MPL exceeding samples was 23%, 22%, and 42% for raw, pasteurized, and sterilized milk samples, respectively (Ghaffarian Bahraman et al., 2020). Moreover, a previous study has reported the prevalence of AFM_1_ contamination in milk samples consumed in continents of Asia (82.6%), Europe (79.1%), Africa (76.4%), and America (41.3%) (Salari et al., 2020).

According to our findings, the AFM_1_ concentration in pasteurized samples (24.89 ng/L) was higher than raw milk samples (13.54 ng/L). Our finding was in line with the results of two Iranian comprehensive meta‐analysis studies indicating a higher AFM_1_ contamination level in processed milk (Ghaffarian Bahraman et al., 2020; Pour et al., 2020). Also, a global meta‐analysis study showed that the overall AFM_1_ concentration in raw milk (57.36 ng/L) was lower than in pasteurized samples (85.39 ng/L) (Mollayusefian et al., 2021). This study, however, reported that AFM_1_ contamination levels of raw milk samples consumed in most included African countries were significantly higher than in European, Asian, and South American countries. This meta‐analysis study also showed that the concentration of AFM_1_ in pasteurized milk samples consumed in Brazil, Mexico, Pakistan, and Syria was higher than in other included countries (Mollayusefian et al., 2021). Several factors affect the contamination level of AFM_1_ in milk depending on the collected‐sample area of origin. Differences in methods of harvesting and storage of animal feed, milking processes, milk transportation, storage, processing, and packaging as well as differences in the method of AFM_1_ detection and quantification are among the most determining factors (Ghaffarian Bahraman et al., 2020; Iqbal et al., 2015).

We also observed that the median concentration of AFM_1_ in raw milk samples collected from the supply level was higher than those collected from milk transport tankers to processing plants. Raw milk distributed in Iranian retail markets is mostly supplied by traditional livestock farms with a small number of animals (1–10 heads). Meanwhile, raw milk delivered to processing plants is mainly supplied by industrial farms with high milk production capacity (Ehsani et al., 2016; Ghaffarian Bahraman et al., 2020). Since small farms are often overlooked in quality control processes, they seem to be able to easily use forage and dry bread contaminated with AFB1 as animal feed (Khoshpey et al., 2011). Furthermore, it is reported that rejected raw milk consignments from processing plants are sold directly to consumers in retail markets due to a lack of adequate supervision (Hashemi, 2016).

Moreover, we found that there was a wide variation in the AFM_1_ contamination levels in different brands of pasteurized milk distributed in the Iranian market (Table 3). The AFM_1_ concentration in the collected samples from brand 11 was more than six times that from brands 1 and 2. Previously, there have been reports about the difference in the AFM_1_ contamination level of pasteurized milk samples from various brands available in the Iranian market (Sani et al., 2010). Seemingly, the chemical structure of AFM_1_ is resistant to the condition of pasteurization and sterilization, so these processes cannot reduce the contamination level of this toxin in raw milk (Ghaffarian Bahraman et al., 2020). For this reason, quality control of forage and animal feed used in livestock farms is the Achilles heel of combating AFM_1_ contamination in dairy products.

Also, we found that the median concentration of AFM_1_ in the samples collected in summer (18.98 ng/L) was not significantly different from those collected in winter (14.67 ng/L). The evidence provides conflicting reports in this regard. Our findings were in line with the results of the study by Tajkarimi et al. which showed that the AFM_1_ contamination of milk samples was not affected by differences in sampling seasons (Tajkarimi et al., 2007). However, some other investigations have found that the milk samples collected in winter were more contaminated in comparison with the summer‐collected samples (Iqbal et al., 2013; Kamkar, 2005; Nemati et al., 2010; Xiong et al., 2021). Researchers of the above studies have attributed this difference to the various nutrition of dairy cattle in the hot and cold seasons. In cold seasons, stored fodder and industrial foods are usually used instead of fresh fodder to feed dairy animals, in which AFB1‐producing fungi are more likely to grow (Mahmoudi & Norian, 2015).

Nonetheless, Iran has faced significant climate change such as rising minimum temperatures and a sharp decline in annual rainfall in recent decades (Daneshvar et al., 2019). In such a situation, it is difficult for dairy farms to provide fresh fodder, even in the spring. Furthermore, Iran has been subjected to severe economic sanctions over the past decade, which has placed restrictions on farmers providing high‐quality animal feed. Therefore, the stored forage is commonly used by industrial and semi‐industrial livestock farms all year round.

Milk consumption is recommended for all age groups due to its beneficial nutritional properties. Children and the elderly are the main audience of these recommendations since they are more vulnerable to nutritional deficiencies (Scholz‐Ahrens et al., 2020). Although not achieving easily, the production of milk completely free of AFM_1_ is considered an ideal goal in dairy industry. Therefore, most countries of the world allow a range of AFM_1_ contamination in milk depending on their specific conditions (Iqbal et al., 2015). Concerns about the presence of excessive amounts of toxins such as AFM_1_ in the milk consumed by the community have prompted health authorities to seek a rational way to assess the risk and quantify the potential consequences of exposure to toxic compounds (Alshannaq & Yu, 2017). In fact, risk assessment can evaluate the effectiveness of existing controlling methods and provide valuable data for adopting effective strategies to deal with toxins' exposure (Paumgartten, 1993). The HI and MoE are two of the most common indicators in assessing the risk of exposure to aflatoxins.

The overall HI value which was calculated using the overall median concentration of the toxin in the current study showed that there was no serious risk of milk consumption in terms of AFM_1_ content (HI <1). The median value of HI in the present study was 0.270, 0.307, and 0.595 for men, women, and children, respectively. However, the HI value in 16%–35% of the individual samples was greater than 1, which indicates a significant risk of postexposure consequences for children and adults of both sexes. Seemingly, the HI value calculated using the mean (median) concentration of toxin cannot solely reflect the actual risk of postexposure health consequences. Therefore, we reported the proportion of individual samples with HI ≤1 and the EU‐MPL Exceeding samples as two side indicators.

In this study, the AFM_1_ exposure risk through milk intake was also assessed using the MoE. The results demonstrated that MoE values were lower than 10,000 for children (male = 14,833, female = 13,044, child = 6719). Therefore, the health risk of exposure to AFM1 through milk consumption was significant in children of this area. Also, the MoE value calculated for individual samples indicated that a percentage of the milk samples were in the alert range in terms of AFM1 contamination level for men (4%), women (4%), and children (18%). The MoE values which provide a comprehensive and accurate risk assessment of AFM1 exposure in human societies are suggested to be reported in future Iranian investigations.

Based on the mean potency estimates of the cancer risk in all age groups, the cancer risk was estimated to be between 0.0003 and 0.0033 cases of HCC per 100,000 person‐years, whereas this value was estimated between 0.0007 and 0.0088 based on the UB potency estimates. In addition, the highest cancer risk was calculated for children. Based on the mean potency estimates for this age class, the cancer risk was estimated to be between 0.0006 and 0.0033 cases of HCC per 100,000 person‐years, whereas this value was estimated between 0.0015 and 0.0088 based on the UB potency estimates. Therefore, the results of the present study also showed that AFM1‐contaminated milk consumption has a small role in increasing the incidence of HCC cancer in southeastern Iran.

## 5 CONCLUSION

This study reports for the first time the exposure risk to AFM_1_ through milk consumption in adults and children in southeastern Iran according to the MoE approach. Considering the health risk assessment indicator of MoE, a significant health risk threatens the child population due to exposure to AFM_1_ through milk consumption in this region. Furthermore, the results of this study showed that the prevalence of AFM_1_‐positive samples (95%) was very high in southeastern Iran. This finding could indicate contamination at different levels of milk production from farm to fork. Therefore, prompt decision‐making to reduce the exposure of people in the community to AFM_1_ seems necessary such as improving livestock conditions and quality control.

## AUTHOR CONTRIBUTIONS

**Ali Ghaffarian‐Bahraman:** Investigation (lead); writing – original draft (lead). **Salman Mohammadi:** Formal analysis (lead); writing – review and editing (equal). **Ali Dini:** Conceptualization (lead); data curation (lead); project administration (lead); writing – review and editing (equal).

## FUNDING INFORMATION

This work was supported by the Rafsanjan University of Medical Sciences [Grant number: 97304].

## CONFLICT OF INTEREST STATEMENT

The authors declare no competing interests.

## Data Availability

The authors confirm that the data supporting the findings of this study are available within the article.
